# Severe preeclampsia: immediate action as a strategy to save lives

**DOI:** 10.61622/rbgo/2025rbgoedt2

**Published:** 2025-10-21

**Authors:** Antonio Braga, Guilherme Ramires de Jesús, Maria Laura Costa, Leandro De Oliveira, José Geraldo Lopes Ramos, José Carlos Peraçoli, Edson Vieira da Cunha, Agnaldo Lopes da Silva, Maria Celeste Osório Wender, Cláudia Mello, Henri Augusto Korkes

**Affiliations:** 1 Universidade Federal do Rio de Janeiro Rio de Janeiro RJ Brazil Universidade Federal do Rio de Janeiro, Rio de Janeiro, RJ, Brazil.; 2 Universidade Federal Fluminense Department of Maternal Child Niteroi RJ Brazil Department of Maternal Child, Universidade Federal Fluminense, Niteroi, RJ, Brazil.; 3 Vassouras University Vassouras RJ Brazil Vassouras University, Vassouras, RJ, Brazil.; 4 Universidade do Estado do Rio de Janeiro Rio de Janeiro RJ Brazil Universidade do Estado do Rio de Janeiro, Rio de Janeiro, RJ, Brazil.; 5 Universidade Estadual de Campinas Department of Gynecology and Obstetrics Campinas SP Brazil Department of Gynecology and Obstetrics, Universidade Estadual de Campinas, Campinas, SP, Brazil.; 6 Universidade Estadual Júlio de Mesquita Filho Department of Gynecology and Obstetrics Botucatu SP Brazil Department of Gynecology and Obstetrics, Universidade Estadual Júlio de Mesquita Filho, Botucatu, SP, Brazil.; 7 Universidade Federal do Rio Grande do Sul Porto Alegre RS Brazil Universidade Federal do Rio Grande do Sul, Porto Alegre, RS, Brazil.; 8 Hospital Moinhos de Vento Obstetrics and Gynecology Department Porto Alegre RS Brazil Obstetrics and Gynecology Department, Hospital Moinhos de Vento, Porto Alegre, RS, Brazil.; 9 Universidade Federal de Minas Gerais Belo Horizonte MG Brazil Universidade Federal de Minas Gerais, Belo Horizonte, MG, Brazil.; 10 Pontifícia Universidade Católica de São Paulo Department of Human Reproduction and Childhood São Paulo SP Brazil Department of Human Reproduction and Childhood, Pontifícia Universidade Católica de São Paulo, São Paulo, SP, Brazil.

Preeclampsia remains as leading cause of maternal mortality worldwide, with 95% of these deaths occurring in low- and middle-income countries.^(1)^ In Brazil, a recent analysis developed in 2023 revealed that preeclampsia accounted for the most important cause of direct obstetric deaths, with a dramatic social and racial disparity: 58.5% of maternal deaths occurred among mixed-race women and 10.57% among black women,^(2)^ underscoring the cruelest expression of inequality in health.

Although prevention of preeclampsia is not yet fully possible, proven interventions can significantly reduce risks: low-dose aspirin in women at risk, calcium supplementation in populations with low dietary calcium-intake, and regular physical activity have been demonstrated to be essential in numerous studies.^(3-5)^ In Brazil, universal calcium supplementation during antenatal care was consolidated in 2025, through a Ministry of Health recommendation,^(6)^ in a pioneering movement initiated by the State Health Secretariat of Rio de Janeiro. This innovative experience was recently reported, reinforcing the relevance of preeclampsia prevention as public health policy.^(7)^ But further progress is urgently needed.

If prevention of preeclampsia remains partial, the immediate treatment of severe features represents the most effective intervention to avoid maternal deaths. In such cases, one of the most important interventions is the early indication of magnesium sulfate (MgSO_4_) for pregnant women with severe preeclampsia or eclampsia. This is a well known, inexpensive, and safe medication, whose benefit has been consistently demonstrated in clinical trials and systematic reviews with meta-analyses. The classic *Magpie Trial* demonstrated that MgSO_4_ halves the risk of seizures in women with preeclampsia,^(8)^ with evidence also supporting its safe use in pre-hospital settings, including primary health care units. Recently, Shields et al.^(9)^ demonstrated that pregnant women with hypertensive crisis, even without symptoms, are benefited by magnesium sulfate administration. A recent *Cochrane* review confirmed that there is no significant difference between administration regimens, demonstrating overall efficacy regardless of route, and reinforcing consistent evidence of safety.^(10)^ Brazilian and international guidelines maintain MgSO_4_ as the gold standard, recommending its immediate initiation upon clinical or laboratory indication.^(11,12)^ Nevertheless, what is commonly observed is that MgSO_4_ is only recommended at maternity hospital and sometimes at tertiary hospitals, highlighting a dangerous delay in adequate assistance.

The clinical criteria that should be promptly considered to indicate MgSO_4_ are readily identifiable in primary care and include: eclampsia, manifested by seizures or loss of consciousness after 20 weeks of gestation; imminent eclampsia, characterized by severe headache, visual disturbances such as scotomas or blindness, or epigastric pain; hypertensive crisis with systolic blood pressure ≥160 mmHg or diastolic ≥110 mmHg, even in the absence of symptoms. In emergency units where laboratory resources are available, additional parameters may strengthen the indication for treatment with MgSO_4_, such as progressive elevation of liver enzymes (AST or ALT >70 IU/L or more than twice the upper limit of normal), thrombocytopenia (platelet count <100,000/mm³) or established HELLP syndrome.^(12)^

It is important to note that MgSO_4_ widely used in two different regimens: the Zuspan regimen, which consists of an intravenous loading dose of 4 g administered over 20 to 30 minutes followed by a continuous maintenance infusion of 1 g per hour, and the Pritchard regimen, which combines an initial intravenous dose of 4 g with 10 g intramuscularly (5 g in each buttock), followed by 5 g intramuscularly every four hours. Both regimens show equivalent efficacy and safety according to the most recent evidence.^(8,13)^

Beyond MgSO_4_, in cases of hypertensive crisis, defined as systolic blood pressure ≥160 mmHg or diastolic blood pressure ≥110 mmHg, the use of rapid-acting antihypertensives is recommended to reduce severe maternal risks without causing abrupt drops that could compromise uteroplacental perfusion.^(12)^ In such cases, oral nifedipine may be given at a dose of 10 mg, repeated every 20 minutes up to a maximum-dose of 30 mg, while intravenous hydralazine may be administered at doses of 5 mg every 20 minutes, up to a maximum-dose of 30 mg. It is important to emphasize that management of hypertensive crisis may commence either with MgSO_4_ or with antihypertensive medications and continuous vigilance is required to reassure maternal and fetal well-being.

Based on this body of evidence, the State of Rio de Janeiro, in partnership with the Specialized National Commission on Hypertension in Pregnancy of the Brazilian Federation of Gynecology and Obstetrics Associations and the Brazilian Network for Studies on Hypertension in Pregnancy (RBEHG), instituted an unprecedented policy: the Orange Code, as part of the VIDA (*Life in English*) initiative – Vigilance, Identification, Diagnosis and Action. This pioneering strategy establishes the mandatory initiation of immediate treatment at all entry points of the health system—primary care units, emergency care units, regional emergency centers, and general hospitals without maternity services, settings where, until now, treatment was often delayed until arrival at the maternity hospital. This initiative has already been implemented in some cities across the country throughout 2024-2025, with the first successful experience taking place in Joinville, as a research protocol (not yet published) or as activities linked to Obstetrics and Gynecology Congresses, with the support of local health departments. The Orange Code also standardizes communication with safe transportation services, mandates minimal documentation for continuity of care, and provides for the implementation of an emergency hypertensive crisis box containing essential supplies for rapid intervention. The *VIDA Regimen* recommends the loading-dose of 4g of MgSO_4_ and prompt progress with the referral process.

Rio de Janeiro is the first Brazilian state to adopt this technical note, but it is hoped that its experience will inspire other states and, above all, that the Ministry of Health will incorporate this initiative as a national policy. Embedded in the current restructuring of maternal and child healthcare, within the framework of the Alyne Network, this intervention has the potential to save thousands of maternal lives and represents a fundamental step for Brazil to achieve the Sustainable Development Goal of reducing the maternal mortality ratio to fewer than 30 deaths per 100,000 live births by 2030.

The fight against preeclampsia is, therefore, a decisive test of the capacity of our health system to provide timely and equitable care. Recognizing urgency, acting without delay, and ensuring continuity of care are not merely technical imperatives: they are ethical commitments to the lives of women.
